# Post-cardiac Injury Syndrome Following Permanent Dual-Chamber Pacemaker Implantation

**DOI:** 10.7759/cureus.21737

**Published:** 2022-01-30

**Authors:** Zeel K Patel, Meet S Shah, Ronak Bharucha, Michael Benz

**Affiliations:** 1 Internal Medicine, Rutgers New Jersey Medical School, Newark, USA; 2 Internal Medicine, University at Buffalo, Buffalo, USA; 3 Interventional Cardiology, Christ Hospital, Jersey City, USA

**Keywords:** etiology of pericarditis, serositis, cardiac temponade, post-cardiac injury syndrome, dressler's syndrome, complications to pacemaker implantation

## Abstract

Post-cardiac injury syndrome is a heterogeneous group of conditions that result from autoimmune-mediated inflammation of the pericardium, epicardium, and myocardium. Interventions such as pacemaker lead insertions, percutaneous coronary interventions, radiofrequency ablations, cardiac surgeries, and Swan-Ganz catheterizations can cause myocardial injury leading to post-traumatic pericarditis. This phenomenon can lead to chest pain, recurrent effusions, and fever along with possible complications of heart failure, arrhythmias, conduction abnormalities as well as cardiac tamponade. Herein, we present a case report of a 64-year-old female with a history of sick sinus syndrome managed with a dual-chamber pacemaker who presented with post-cardiac injury syndrome after three months of pacemaker implantation. She developed a recurrent syndrome of fever, chest discomfort, tachycardia with weakness, hemodynamic instability, hemorrhagic serositis, and cardiac tamponade. The mechanism of exudative inflammatory effusions initially remained inconclusive, as the workup for infectious and malignant processes was negative. However, post-cardiac injury syndrome akin to the Dressler syndrome related to screw-in dual-chamber pacemaker implantation remained a possibility. Her condition was acutely managed with a combination of colchicine and glucocorticoid therapy. She was placed on long-term aspirin and colchicine therapy to prevent any recurrences. This article illustrates a case of post-cardiac injury syndrome after dual-chamber pacemaker implantation, including details of evaluation, management, complications and monitoring of patient progress.

## Introduction

The myocardium of the heart is anatomically surrounded by a sac of fibroelastic tissue known as the pericardium. It consists of two layers of membranous tissue known as the parietal and visceral layers. A potential space exists between these two layers, which physiologically holds an average of 15 to 35 mL of pericardial fluid to lubricate against the motion of the heart [1]. Pericarditis, inflammation of the pericardium, can be caused by multiple factors but has a typical clinical presentation namely, fever, pleuritic chest pain, and tachycardia with a pericardial friction rub on physical exam. During inflammation, the friction between the heart and the pericardium can induce chest pain, oftentimes mimicking the pain of a heart attack. An increase in white blood cell count, inflammatory markers like erythrocyte sedimentation rate or C-reactive protein are typically seen along with ECG changes such as diffuse ST-segment elevation with PR depression. According to the American Heart Association, the etiology of pericarditis can be infectious (viral, bacterial, fungal), autoimmune (lupus, scleroderma, rheumatoid arthritis), or malignant. A form of secondary pericarditis that occurs as a result of injury to the heart or pericardium after a myocardial infarction is termed Dressler syndrome (DS), first described by Dr Dressler in 1956. Chest radiograph showing cardiomegaly in relevant clinical scenarios can also raise clinical suspicion for DS. It is further classified as a component of other syndromes under the umbrella term post-cardiac injury syndromes. A previous case report cited that only nine cases of post-cardiac injury syndrome following pacemaker insertion were documented dating back to 1975 [2].

While the exact pathophysiology of Dressler syndrome is unknown, it is currently thought to be an immune-mediated mechanism. A proposed theory is that injury to mesothelial pericardial cells induces an immune response, leading to immune complex deposition in the pericardium, pleura, and lungs, which causes an inflammatory response [3]. This syndrome occurs with a latency period of weeks to months following cardiac or pericardial injury before the development of pericardial effusion, rarely occurring earlier than three weeks post-injury. Some of the risk factors for developing this syndrome are myocardial infarction, viral infections, cardiac surgery involving the myocardium, pericardium or the epicardium, trauma, prior history of pericarditis, prior treatment with prednisone, and use of halothane anesthesia [3]. In addition, there have been cases of DS being reported after transvenous pacemaker implantation, permanent pacemaker implantation, and coronary angioplasty.

The incidence of post-cardiac injury syndrome has largely decreased in the era of reperfusion therapies post-myocardial infarction. In a cohort of patients treated with fibrinolysis after myocardial infarction, only one of 201 patients developed post-cardiac injury syndrome [4]. In another study of 28,761 patients undergoing cardiothoracic surgery, only 1.7% (493 patients) developed post-cardiac injury syndrome requiring hospital admission or contributing to mortality [5].

The treatment of Dressler syndrome consists of similar treatment to other cases of acute pericarditis. The first-line therapy includes a combination of non-steroidal anti-inflammatory drugs and colchicine. Post-operative colchicine given prophylactically for 30 days has been shown to significantly reduce the incidence of post-cardiac injury syndrome after cardiac surgery [6]. If the patient develops pericardial effusion leading to cardiac tamponade, treatment with a pericardial window or surgical drainage may be necessary. Herein, we present a case of a female patient with a history of bradycardia-tachycardia syndrome treated with permanent pacemaker implantation, who displayed multiple symptoms of post-cardiac injury syndrome.

## Case presentation

**Figure 1:**
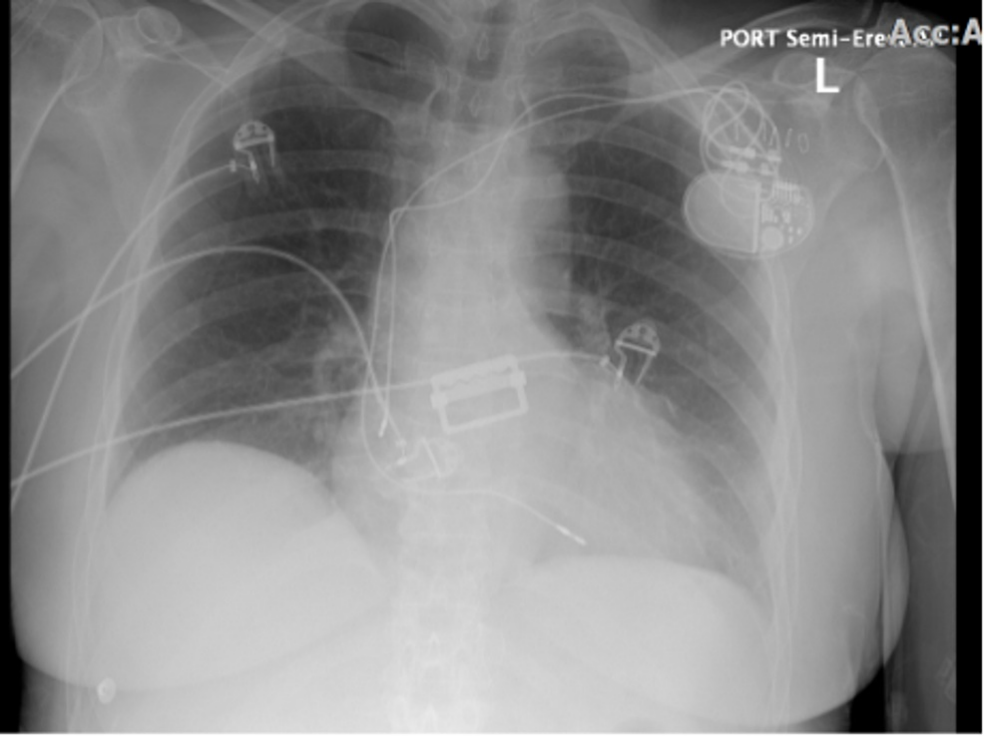
Chest X-ray showing proper placement of dual-chamber leads after pacemaker implantation

**Figure 2:**
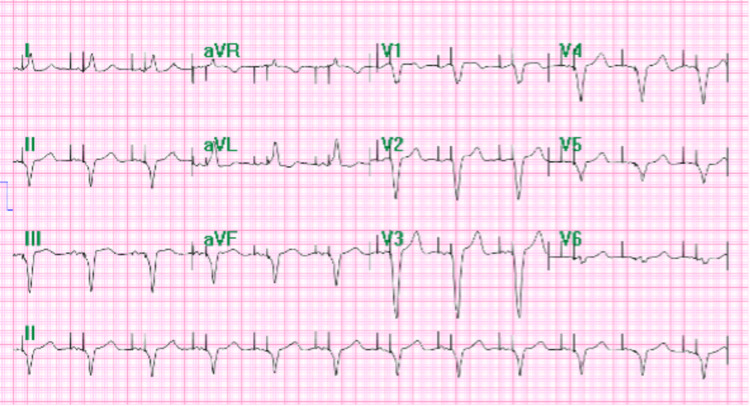
ECG tracing showing an atrioventricular dual-paced rhythm suggesting a properly functioning pacemaker after its implantation

A 64-year-old Caucasian female with a past medical history of bradycardia-tachycardia syndrome, hypertension, and osteoporosis presented to the emergency department (ED) with chief complaints of generalized weakness, dizziness near syncope, and severe migraine. Further workup revealed hypotension, atrial fibrillation with a rapid ventricular response, and anemia (current hemoglobin at 8 g/dL from her baseline 14 g/dL) with no rectal bleed, hematemesis, and hematochezia or melena. Surgical history includes hysterectomy, thyroid surgery, lung mass biopsy (benign results) and a permanent dual-chamber pacemaker that was implanted three months ago for sick sinus syndrome (Figures 1, 2). Her medications include apixaban, metoprolol, and enalapril. The patient worked at a shipping company and admitted to mild alcohol use but denied any tobacco or illicit drug usage. In the ED, she received blood transfusions and was started on digoxin for hemodynamic instability unresponsive to IV fluids. Home medications were subsequently discontinued due to a positive fecal occult blood test. Her labs showed albumin 3.6, elevated international normalised ratio (INR) 1.4 of unclear etiology with elevated blood urea nitrogen (BUN) 24 and creatinine 1.3.

**Figure 3:**
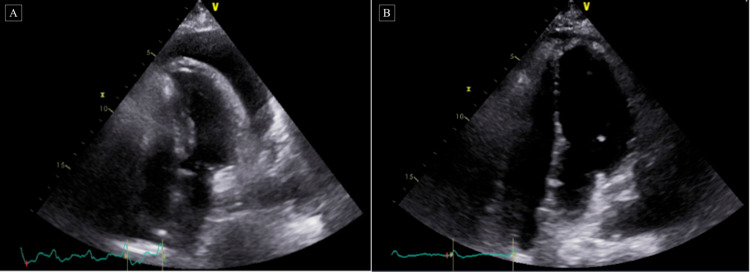
Echocardiograms before and after corticosteroids therapy A: Limited echocardiogram to evaluate for pericardial effusion. Large circumferential pericardial effusion with the diastolic collapse of the right ventricle and respiratory variation of mitral inflow consistent with cardiac tamponade. B: Limited follow up echocardiogram following steroids therapy after pericardial window. Small pericardial effusion essentially resolved compared to prior echocardiogram.

**Figure 4:**
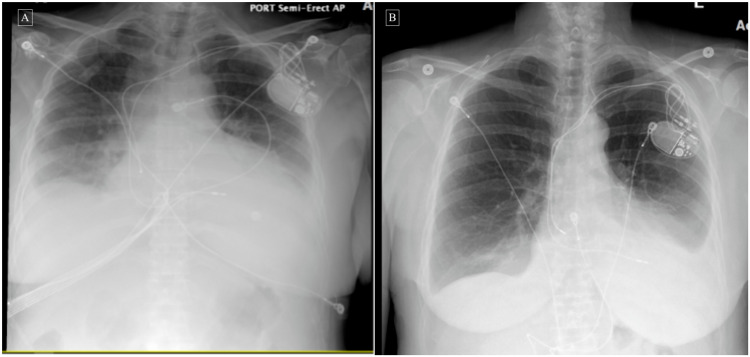
Chest X-rays before and after corticosteroid therapy A: Chest X-ray showing left-sided pleural effusion and/or atelectasis/infiltrate, right-sided pleural effusion, and right basilar atelectasis. B: Chest X-ray after steroids therapy showing decreased left-sided pleural effusion with improved aeration at the left lung base with mild left basal compressive atelectasis and residual effusion, and mild cardiomegaly.

Before her upper endoscopy procedures, the patient developed hypotension, tachycardia, and hypoxemia with 85% O2 saturation. The procedure was cancelled, and she was subsequently transferred to ICU to be stabilized. On the following day, she developed another episode of hypotension, tachycardia with weakness, abdominal pain and shortness of breath. A bedside echocardiogram (Figure 3) revealed cardiac tamponade for which she underwent immediate pericardial window with a mediastinal drain. Her pericardial histologic studies showed chronic inflammation with reactive changes, but they were negative for acid-fast bacilli and malignant cells. Cytology of pericardial fluid revealed hemorrhagic fluid with RBCs and WBCs. Further workup revealed iron deficiency anemia, which was treated with IV iron. She also had an abdominal ultrasound and CT abdomen/pelvis, which ruled out any liver pathology like cirrhosis or other intrabdominal processes. Over the next few days, she continued to have recurrent episodes of tachycardia with weakness that was treated with carvedilol. One morning, she developed sudden onset chest discomfort, pain and substernal pressure after waking up. An immediate ECG and cardiac enzymes were negative for acute coronary syndrome, and a chest X-ray showed bilateral pleural effusions (Figure 4). A lead perforation was unlikely given the time frame of presentation, no obvious signs on echocardiogram and chest x-ray, and the presence of bilateral pleural effusions. As her condition improved and became hemodynamically stable, she underwent esophagogastroduodenoscopy (EGD) and colonoscopy. Biopsy results showed chronic active gastritis in the gastric body with negative results for *H. pylori,* trichrome stain for parasites, and periodic acid-Schiff (PAS) positive inclusions in macrophages. Colonoscopy revealed non-bleeding internal hemorrhoids.

After a few days of stabilization and relative improvement in symptoms, she had another episode of tachycardia, squeezing chest pressure, and hypotension. Due to a high suspicion of pulmonary embolism and a positive D-dimer test, she had a chest CT with an angiogram. While it was negative for pulmonary embolism, it revealed an increased left-sided pleural effusion. She underwent ultrasound-guided thoracentesis draining 800 cc serosanguineous fluid. She began spiking low-grade fevers without an infectious source over the next few days. Given her recurrent hemorrhagic serositis on a background of unremarkable past medical history, paroxysmal atrial fibrillation with anticoagulation, and pacemaker implantation, circumstantial evidence raised suspicion for post-cardiac injury syndrome akin to the Dressler syndrome. As a result, she was started on a trial of colchicine 0.5 mg twice a day but showed minimal improvement and continued to have episodes of tachycardia with weakness and persistent pleural/pericardial effusions that were tracked by chest X-ray and chest CT.

Due to increasing pleural effusion seen on her chest CT, she underwent another thoracentesis yielding 1100 cc amber-colored fluid. After excluding any underlying infectious etiology, she was started on a trial of IV methylprednisolone 40 mg daily along with colchicine. Her condition drastically improved after beginning steroids therapy and follow up chest X-ray/echocardiogram showed decreasing pleural effusion and pericardial effusion compared to prior studies (as seen above in Figures 3, 4). Her vitals remained stable with improved symptoms of fever, chest pain, palpitations, weakness, and dyspnea. The workup for infectious or autoimmune etiology causing her effusions, including HIV, Lyme disease, herpes simplex virus (HSV), rapid plasma reagin (RPR), echovirus, cytomegalovirus (CMV), QuantiFERON gold and QuantiFERON gold plus, coxsackievirus A/B, fungal culture, antinuclear antibodies (ANA), rheumatoid factor, and ceruloplasmin, was negative. With appropriate clinical improvement and a chest X-ray showing mild left lower lung pleural effusion, she was discharged with prednisone 60 mg tapered down over two weeks and a 15-day course of colchicine 0.5 mg twice a day.

One month later, the patient presented again in the ED with symptoms of generalized weakness, fevers, chills, headache, and tachycardia. Due to the presence of leukocytosis, she was started on antibiotics for sepsis. A chest CT without contrast revealed a consolidation at the left lung base representing either pneumonia or atelectasis accompanied by mild cardiomegaly, pericardial effusion, and bilateral pleural effusions. With rising fevers and a two-day course of antibiotics, a CT scan of the abdomen and pelvis revealed no abnormalities. Additionally, an echocardiogram revealed a small, pericardial effusion with no changes compared to her previous visit. Workup for infectious or malignant processes did not reveal a clear etiology of her symptoms. Due to an unknown etiology of her recurrent symptoms, she was transferred to a tertiary care facility for further workup and management.

Follow-up after discharge from the tertiary care institution

The patient underwent an extensive workup that did not reveal a clear etiology of her syndrome. She was placed on aspirin 650 mg three times a day tapered over several weeks and long term colchicine 0.5 mg twice a day, which she tolerated well without further recurrences of her symptoms.

## Discussion

Post-cardiac injury syndrome is a heterogeneous group of autoimmune-mediated conditions that cause inflammation to either the pericardium, epicardium, or myocardium. The principal conditions under this category include post-pericardiotomy syndrome, post-traumatic pericarditis, and post-myocardial infarction syndrome. Collectively, it is presumed that initial injury to pericardial cells combined with blood in the pericardial space triggers an immune response, resulting in immune complex deposition in the pericardium, pleura, and lungs [3]. This hypothesis is supported by the evidence of discrete latent period between cardiac injury and clinical onset of symptoms of weeks to months, coexistent pleural effusion and possible pulmonary infiltrates, and increased anti-cardiac antibodies [7,8]. Previous studies have shown a statistically significant correlation between the pre and post-operative ratio of anti-actin and anti-myosin antibodies and clinical occurrence of post-cardiac injury syndrome [7,8]. While post-myocardial infarction syndrome (Dressler syndrome) is the prototype of post-cardiac injury syndrome, it can also occur as a complication of cardiac surgery (post-pericardiotomy syndrome) or cardiac trauma (post-traumatic pericarditis).

This atypical case of post-cardiac injury syndrome in a 64-year-old woman with a history of bradycardia-tachycardia syndrome and dual-chamber pacemaker implantation illustrates the presence of post-pacemaker implantation pericarditis. As a rare form of post-cardiac injury syndrome with an estimated incidence of 1% to 2% after pacemaker implantation, post-pacemaker implantation pericarditis is similar to the Dressler syndrome that typically presents with symptoms one to six weeks following initial pericardial damage [9]. Other predisposing factors for DS include myocardial infarction, viral infections, cardiac surgery involving the myocardium, pericardium or the epicardium, trauma, prior history of pericarditis, prior treatment with prednisone, and use of halothane anesthesia [3]. Patients usually experience fever, malaise, pleuritic chest pain, irritability, decreased appetite, palpitations, tachycardia, dyspnea, and arthralgias. In addition, some patients may even exhibit signs of pneumonitis, ranging from no pulmonary complaints to significant respiratory distress with large pulmonary effusions. This patient’s hemorrhagic pericardial effusion was initially believed to be a result of coagulopathy secondary to apixaban treatment for atrial fibrillation until further work-up was performed.

The standard diagnostic procedure and most sensitive imaging study for evaluating a patient with suspected Dressler syndrome is an echocardiogram. An echocardiogram will allow for evaluation of the pericardial fluid and ventricular contractility. In addition, a cardiac MRI may also be used to determine whether there is an effusion present. In a patient with DS, an ECG will also initially demonstrate global ST-segment elevation and T-wave inversion similar to acute pericarditis. If there is a large volume pericardial effusion present, both electrical alternans and/or a low voltage QRS may be observed. Thus, the diagnosis of post-cardiac injury syndrome is typically suspected based upon the clinical findings of pleuritic chest pain and fever in a patient with myocardial infarction or pericardial injury confirmed by laboratory testing, ECG, chest radiograph, and echocardiogram findings.

Most patients with suspected post-cardiac injury syndrome are typically treated with non-steroidal anti-inflammatory drugs (NSAIDs)/aspirin and colchicine, which are tapered over weeks as the symptoms resolve [10]. Patients who do not tolerate NSAIDs and colchicine therapy or have a resolution of symptoms may be given a course of corticosteroids [10]. Our patient was placed on only colchicine therapy initially due to the bleeding risks associated with NSAIDs. She failed to show adequate improvement on colchicine alone and therefore, she was treated with a trial of corticosteroids, which resulted in marked improvement of her condition.

The prognosis for patients with post-cardiac injury syndrome is typically considered to be quite positive. Patients requiring pericardial drainage usually have a favorable prognosis. However, they also have an increased risk for reaccumulation of fluid and subsequent need for repeat pericardiocentesis and adjustments of medication regimens.

Post-pacemaker implantation pericarditis is a rare form of post-cardiac injury syndrome that can be managed appropriately if identified early in the course of the disease. Delayed diagnosis can potentially result in more serious complications, such as cardiac tamponade. While our patient did not show adequate improvement with only colchicine treatment, she was responsive to a combination of steroids and colchicine treatment. Ultimately, she was placed on aspirin and colchicine treatment to prevent any recurrence of her condition. Due to her modest CHADS2-VASC (congestive heart failure, hypertension, age ≥ 75 years, diabetes mellitus, stroke or transient ischemic attack, vascular disease, age 65 to 74 years, sex category) score and a possibility of recurrent bleeding, it was deemed necessary to maintain her on aspirin and discontinue anticoagulation until further clearance from gastroenterology (GI) specialists. 

## Conclusions

This atypical case of permanent pacemaker implantation induced pericarditis demonstrates an uncommon but possible phenomenon of post-cardiac injury syndrome. The diagnosis of post-cardiac injury syndrome following screw-in dual-chamber pacemaker implantation was supported only after excluding other common etiologies of infectious, autoimmune, or malignant in nature. Thus, when a patient presents with the signs and symptoms of pericarditis, it is not only important to look at their medical history but procedural history in temporally relevant cases can also give crucial clues to an appropriate diagnosis. Ultimately, the treatment of post-cardiac injury syndrome typically involves NSAIDs and colchicine. However, a minority of patients with refractory symptoms or various complications may require treatment with systemic glucocorticoid therapy in combination with colchicine, as seen with our patient. 
